# A MEMS Resonant Sensor to Measure Fluid Density and Viscosity under Flexural and Torsional Vibrating Modes

**DOI:** 10.3390/s16060830

**Published:** 2016-06-06

**Authors:** Libo Zhao, Yingjie Hu, Tongdong Wang, Jianjun Ding, Xixiang Liu, Yulong Zhao, Zhuangde Jiang

**Affiliations:** State Key Laboratory for Manufacturing Systems Engineering, Collaborative Innovation Center of Suzhou Nano Science and Technology, Xi’an Jiaotong University, Xi’an 710049, Shaanxi, China; hyj8880@foxmail.com (Y.H.); wtdwxy@163.com (T.W.); dianjianjun@126.com (J.D.); leoxixiang@foxmail.com (X.L.); zhaoyulong@mail.xjtu.edu.cn (Y.Z.); zdjiang@mail.xjtu.edu.cn (Z.J.)

**Keywords:** MEMS resonant sensor, microcantilever, density and viscosity, measuring accuracy, vibrating mode

## Abstract

Methods to calculate fluid density and viscosity using a micro-cantilever and based on the resonance principle were put forward. Their measuring mechanisms were analyzed and the theoretical equations to calculate the density and viscosity were deduced. The fluid-solid coupling simulations were completed for the micro-cantilevers with different shapes. The sensing chips with micro-cantilevers were designed based on the simulation results and fabricated using the micro electromechanical systems (MEMS) technology. Finally, the MEMS resonant sensor was packaged with the sensing chip to measure the densities and viscosities of eight different fluids under the flexural and torsional vibrating modes separately. The relative errors of the measured densities from 600 kg/m^3^ to 900 kg/m^3^ and viscosities from 200 μPa·s to 1000 μPa·s were calculated and analyzed with different microcantilevers under various vibrating modes. The experimental results showed that the effects of the shape and vibrating mode of micro-cantilever on the measurement accuracies of fluid density and viscosity were analyzed in detail.

## 1. Introduction

The density and viscosity of fluid are the most important parameters in the oil [1,2], chemical [3] and medical [4] industries and so on. The density can be measured based on the resonant frequency shift of resonant devices [5]. Reference [6] showed that a microresonator had different resonant frequencies and quality factors due to different densities and viscosities of the loaded liquids. Generally, the resonant frequency of the microresonator decreased with increasing liquid density, and the full width at half maximum (FWHM) of the microresonator increased with increasing liquid viscosity [7,8]. With the development of micro electromechanical systems (MEMS) technology, the MEMS resonator has higher resonant frequency and quality factor, and presents many advantages to implement online measurements of fluid parameters, such as fast response times [9,10].

On the basis of the above phenomena, many MEMS resonant sensors have been studied for the measurement of fluid density and viscosity. Oden [11], Ahmed [12], and Papi [13] studied the measurement of fluid viscosity using the resonant frequency shift of an Atomic Force Microscope (AFM) microcantilever before and after being submerged in the fluid, but the high cost and special installation requirements of the AFM make this method difficult to use in industry. Goodwin [14,15] developed a rectangular silicon microcantilever with a silicon on insulator (SOI) wafer to measure fluid density and viscosity through a vibrating method, and a large number of experimental data of fluid density and viscosity were given in those papers. The measuring accuracies of this sensor for viscosity and density were 10% and 1%, respectively, when the fluid viscosity was less than 1 mPa∙s. The sensor also had lower measurement sensitivity because the piezoresistors were fabricated using polysilicon with a low piezoresistive coefficient. The quality factor of a rectangular microcantilever was significantly reduced with the increase of fluid viscosity due to the increase of resonator energy dissipation, so this type of sensor could not be used to measure high viscosity fluids. Boudjiet [16] studied the effects of microcantilever shape and geometrical dimensions on the density sensitivity. This study showed that a wide and short cantilever was more sensitive to the density variation, the highest sensitivity was 228 Hz/(kg·m^−3^) and the measurement accuracy ranged from 0.4% to 0.6%. To improve the sensitivity of microcantilever sensors, Ansari [17] analyzed and compared the deflections and vibration characteristics of rectangular and trapezoidal profile microcantilevers. The results showed that the trapezoidal microcantilever had better sensitivity. To improve the quality factor of the resonator, Lucklum [18] designed a density and viscosity sensor, where a vibration plate was supported by four elastic beams and driven by Lorentz force undergo a reciprocating in-plane motion. The sensor could measure the square root of the product of density and viscosity with an accuracy of 1% in the range of 1~500 mPa∙s, but the density and viscosity were not measured separately. When the microcantilever was used to detect ultrasensitive mass, an optimized electromagnetic excitation method specifically for the second resonant mode was proposed and developed for further improving the resolution [19]. In conclusion, the resonant sensors reported in the literature above mainly operate under the first order resonant mode, which is also called the flexural resonant mode. Recently, Manzaneque [20] designed a piezoelectric MEMS resonator to measure fluid viscosity and density based on the second order bending mode, the measurement ranges of viscosity and density were 0.4–7.3 mPa∙s and 680–905 kg·m^−3^, and the measuring accuracies of viscosity and density were 8% and 0.4%, respectively. All this suggests that the performance of resonant sensors under higher resonant modes should be researched further.

In this paper, the theories of fluid density and viscosity measurement using the microcantilever resonator were analyzed in detail. Sensing chips with micro-cantilevers in two different shapes were designed via numerical analysis. The MEMS resonant sensor was fabricated with the sensing chips by the packaging process. The first two order resonant modes of micro-cantilevers were both used to measure densities and viscosities of different fluids. The experimental results were analyzed to discuss the effects of the microcantilever structure and the resonant modes on the accuracies of the density and viscosity measurements. Compared with the reference values, the sensor had higher accuracy to measure the fluid viscosity under the higher order resonant mode, but had approximate accuracies to measure the fluid density under the first two resonant modes.

## 2. Theory and Simulation

**Figure 1:**
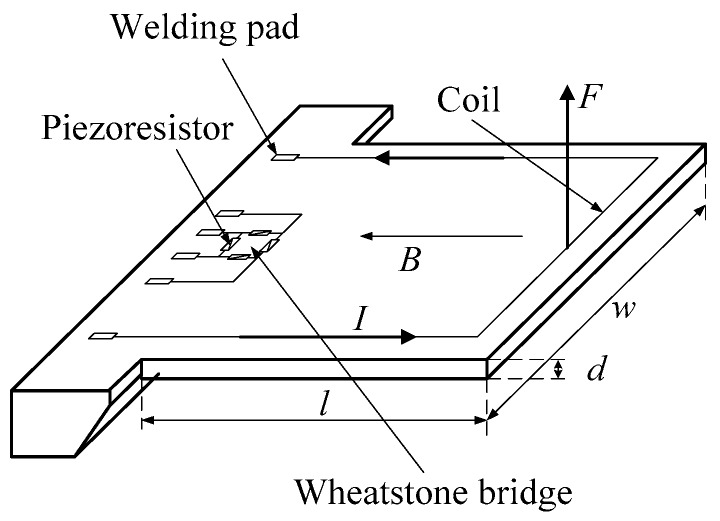
Schematic diagram of the micro-cantilever resonant sensor.

The schematic diagram of the microcantilever resonant sensor is shown in Figure 1. The micro-cantilever was designed with a length *l*, width *w* and thickness *d*. The metal coil was positioned on the surface of the micro-cantilever. The Wheatstone bridge consisted of four piezoresistors designed to be located at the positions of stress concentration. Welding pads were used to connect with the input and output signals. *B* denotes the constant magnetic intensity provided by the external magnet. When the metal coil was powered with the alternating current *I* in a certain frequency, an alternating Lorentz force *F* was generated to drive the microcantilever to vibrate at the same frequency. Then, the resistance values of four piezoresistors were changed based on the piezoresistive effect because the stress conditions of the four piezoresistors varied. The Wheatstone bridge would output the corresponding signal which was proportional to the magnitude of the microcantilever vibration. Therefore, a dynamic resonant curve was obtained from the output of the Wheatstone bridge. When the frequency of the alternating current through the metal coil was close to the natural frequency of the microcantilever, then the microcantilever would vibrate in resonance. A peak value of the Wheatstone bridge output signal appeared in the resonant curve, then the resonant frequency and quality factor of the micro-cantilever could be calculated.

The fundamental frequencies *f*_1_ and *f*_2_ of the microcantilever under the first order flexural mode and the first order torsional mode can be expressed by Equations (1) and (2), respectively [21].

(1) $$f_{1}=\frac{d}{4\pi }\frac{{\phi_{1}}^{2}}{l^{2}}\sqrt{\frac{E}{3\rho_{c}}}$$

(2) $$f_{2}=\frac{1}{4l}\sqrt{\frac{G\xi }{\rho_{c}J_{P}}}$$

where *φ*_1_ is the first order positive root of equation 1 + cosh(*φ_n_*)cos(*φ_n_*) = 0, *φ*_1_ = 1.8751. *ρ_c_* and *E* are the density and elasticity modulus of micro-cantilever, respectively. *G* is the shear modulus, and *G* = *E*/(2 + 2*ν*), where *ν* is the Poisson ratio. *J_p_* is polar moment of inertia and *J_p_* = (*wd*^3^ + *w*^3^*d*)/12. *ξ* can be expressed as follows [22], where *n* is the order number:

(3) $$\xi =\frac{1}{3}d^{4}\left(\frac{w}{d}-\frac{192}{\pi^{5}}\sum_{n=1}^{\infty }\frac{1}{n^{5}}\tanh\frac{n\pi w}{2d}\right)$$

### 2.1. Measurement Theory

The different measurement equations for the fluid density and viscosity were analyzed under different conditions as follows: some assumptions should be discussed first. In general, the microcantilever was assumed as an isotropic linearly elastic solid. The internal frictional effect was negligible and the vibration amplitude was far smaller than the length scale of the micro-cantilever in geometry. The fluid can be considered to be inviscid in practical application when the Reynolds number (Re) >> 1 [23].

The resonant frequency and half peak width of the microcantilever have close relationships with fluid density and viscosity when the microcantilever vibrates resonantly in the measured fluids. The relationship of the resonant frequency and fluid density can be described as follows under the flexural vibration [24]. This approximation is implemented with good accuracy in the derivation of the well-known inviscid result for a rectangular cantilever [23].

(4) $$\frac{f_{\text{fluid1}}}{f_{\text{vac1}}}={\left(1+\frac{\pi \rho_{f}w}{4\rho_{c}d}\right)}^{-1/2}$$

where *f*_fluid1_ and *f*_vac1_ are the flexural resonant frequencies of the microcantilever in the fluid and vacuum, respectively. *ρ*_f_ is the density of the fluid.

The relationship between the resonant frequency and fluid density under the torsional vibration can be described as follows [25]:

(5) $$\frac{f_{\text{fluid2}}}{f_{\text{vac2}}}={\left(1+\frac{3\pi \rho_{f}w}{32\rho_{c}d}\right)}^{-1/2}$$

where *f*_fluid2_ and *f*_vac2_ are the torsional resonant frequencies of micro-cantilever in the fluid and vacuum, respectively.

Equation (4) can be expressed in another form:

(6) $$\rho_{f}=\frac{4\rho_{c}d}{\pi w}\left(\frac{f_{\text{vac}1}^{2}}{f_{\text{fluid}1}^{2}}-1\right)$$

In addition, Equation (5) can be also expressed as follows:

(7) $$\rho_{f}=\frac{32\rho_{c}d}{3\pi w}\left(\frac{f_{\text{vac}2}^{2}}{f_{\text{fluid}2}^{2}}-1\right)$$

In Equations (6) and (7), *f*_vac1_, *f*_vac2_, *ρ*_c_, *d* and *w* all need to be determined before the fluid density is measured via *f*_fluid1_ and *f*_fluid2_. However, they are all constants for a specific microcantilever.

Equations (6) and (7) were deduced for a microcantilever with a length much larger than its width (*l*/*w* >> 1). In addition, [14] has discussed the fluid density and viscosity measurement method with a cantilever plate structure (*l*/*w* ≈ 1), where the relationship of the resonant frequency and fluid density could be expressed as follows:

(8) $$\rho_{f}=\frac{E\upsilon_{n}^{5}d^{3}}{24\left\{1-\sigma^{2}\right\}l^{5}{\left(2\pi f_{\text{fluid}}\right)}^{2}}-\frac{\rho_{c}d\upsilon_{n}}{2l}$$

where *σ* is Poisson’s ratio, *υ_n_* is the eigen value of the fluid velocity potential function. In Equation (8), all variables except *ρ*_f_ and *f*_fluid_ are constants for a specific micro-cantilever.

For Re >> 1, the frequency response of a rectangular cantilever in a viscous fluid is given by [26]:

(9) $$\frac{f_{\text{fluid}}}{f_{\text{vac}}}={\left(1+\frac{\pi \rho_{f}w}{4\rho_{c}d}\mathrm{Real}\left[\Gamma (f_{\text{fluid}})\right]\right)}^{-1/2}$$

where Real [Γ(*f*_fluid_)] is the real part of the hydrodynamic function evaluated at the frequency in the liquid. However, the dimensions of the resonator in [26] are 2.8 mm × 2.6 mm, indicating that Equation (9) can be rearranged as Equation (10) due to the Real [Γ(*f*_fluid_)] approaches 1 at Re >> 1:

(10) $$\rho_{f}=\frac{4\rho_{c}d}{\pi w}\left(\frac{f_{\text{vac}}^{2}}{f_{\text{fluid}}^{2}}-1\right)$$

Therefore, Equations (6)–(8) and (10) can be all simplified as the following Equation (11) since the parameters of cantilever itself can be all confirmed:

(11) $$\rho_{f}=\frac{k_{1}}{f_{\text{fluid}}^{2}}+k_{2}$$

where *k*_1_ and *k*_2_ are constants and calculated by experimental calibrations. However, both *k*_1_ and *k*_2_ are different under flexural and torsional resonant modes. Therefore, this paper used Equation (11) as working equation to measure the fluid density under different conditions such as the flexural and torsional resonant modes.

The resonance quality factor *Q* is an important parameter for fluid viscosity measurement. When a resonator with random shape oscillates in a liquid, the inertial and viscous forces apply to the motion [27], and *Q* is given by:

(12) $$Q^{2}\propto {\left(2\pi f_{\text{fluid}}\right)}^{3}\eta_{f}\rho_{f}$$

where *η*_f_ is the viscosity of the liquid.

Based on the above viewpoint, the relationship of the resonant frequency and fluid viscosity can be described as [14]:

(13) $$\eta_{f}=\frac{k_{3}}{\rho_{f}f_{\text{fluid}}^{3}}{\left(\frac{2g_{\text{fluid}}}{f_{\text{fluid}}}-\frac{2g_{\text{vac}}}{f_{\text{vac}}}\right)}^{2}$$

where *k*_3_ is a constant and obtained by the experimental calibration, *g*_fluid_ and *g*_vac_ are half peak widths of the microcantilever in the fluid and vacuum, respectively. For a specific micro-cantilever, *f*_vac_ and *g*_vac_ are also constants and can be determined via calibration, so the ratio of *g*_vac_/*f*_vac_ can be replaced by *k*_4_. Then Equation (13) becomes:

(14) $$\eta_{f}=\frac{k_{3}}{\rho_{f}f_{\text{fluid}}^{3}}{\left(\frac{2g_{\text{fluid}}}{f_{\text{fluid}}}-k_{4}\right)}^{2}$$

### 2.2. Fluid-Structure Interaction Simulation

Microcantilevers with rectangular and trapezoidal shapes were proposed. The design dimensions of the two different microcantilevers were a length of 1500 μm, width of 2500 μm, thickness of 20 μm, and the free end width of the trapezoidal micro-cantilever was 1000 μm. It is proven that a microcantilever with a larger width could help to improve the sensitivity [16] and quality factor [28] of a microcantilever, and in particular it could improve the Re (Re = *ρ*_f_*fw*/4*η*_f_), where *f* is the resonant frequency of the microcantilever) [23]. If The Re is much larger than 1, it means the viscosity equation and the density equation could be decoupled when the fluid density and viscosity are measured simultaneously. In this study, the Re of the proposed micro-cantilever was about 100, so the resonant frequency of microcantilever was only affected by the fluid density as shown in Equation (11), and the viscosity was calculated by the measured density, quality factor and resonant frequency of the micro-cantilever in the fluid, as shown in Equation (14).

**Figure 2:**
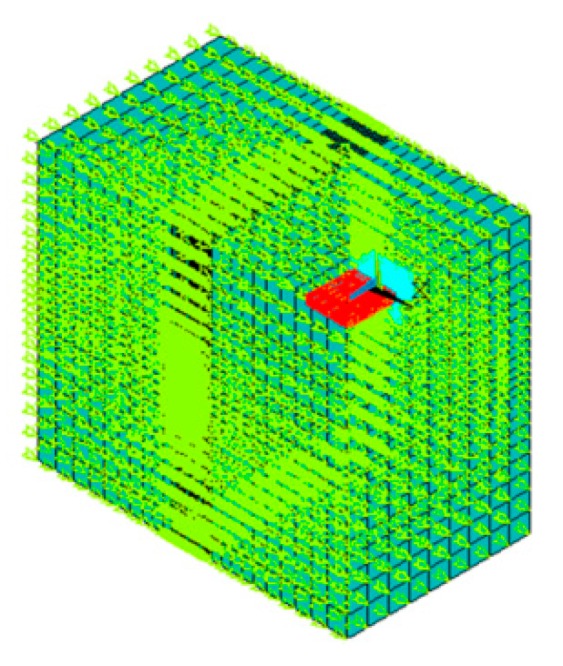
Fluid-solid coupling model.

Fluid-solid coupling simulations were carried out by the finite element method (FEM) as shown in Figure 2. The silicon microcantilever was modeled using the SOLID45 solid element (the red part), its elastic modulus was 169 GPa, its density was 2330 kg/m^3^, and the Poisson’s ratio was 0.064. The fluid was described by the FLUID30 acoustic fluid element (the green part), and the fluid elements were coupled with solid elements. The fluid domain covered the microcantilever except for the fixed end, and the dimension of the fluid domain was increased by 5000 μm over the dimensions of the microcantilever. The mapping method was used to mesh the structure with the number of solid elements, which was 300, and the number of fluid elements which was 2000. The resonant frequency *f* was obtained by modal analyses in the fluid-solid coupling simulation. However, the quality factor *Q* has not been obtained from the current simulation because the peak width at half height cannot be calculated by the simulation results.

**Figure 3:**
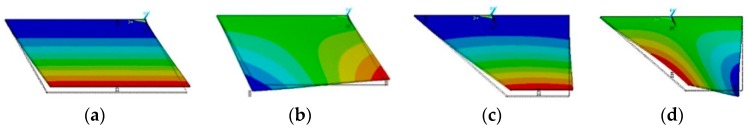
Vibration shapes of two micro-cantilevers in deflection under the first two order modes. (**a**) Rec.1st; (**b**) Rec.2nd; (**c**) Tra.1st; (**d**) Tra.2nd.

The resonant modes of different micro-cantilevers were obtained by fluid-solid coupling simulation in *n*-pentane, as shown in Figure 3, the resonant modes in other fluids were also obtained using the same method. The first order mode was the first order flexural vibration, and the second order mode was the first order torsional vibration in all different fluids. Figure 3a,b showed that the vibrating shapes of the rectangular micro-cantilever under the first and second order modes, respectively. Figure 3c,d reveal the vibrating shapes of the trapezoidal microcantilever under the first and second order modes, respectively. Thus, it could be seen that the vibrating shapes of the microcantilever obviously has nothing to do with the fluid environment, but are mainly related to the microcantilever structure (the length-width ratio).

**Figure 4:**
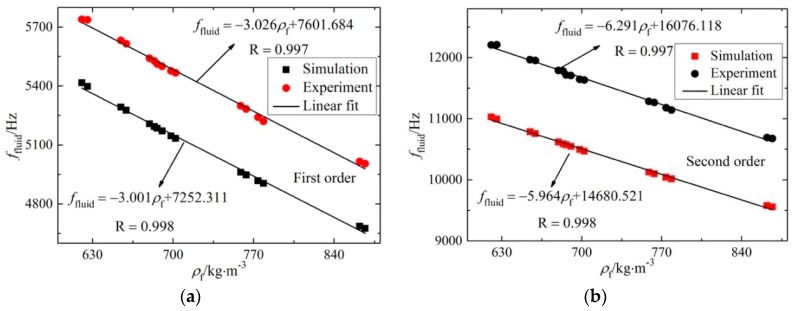
The relationship of resonant frequency and density under different order modes. (**a**) Under the first order mode (flexure); (**b**) under the second order mode (torsion).

The rectangular micro-cantilever was taken as an example to analyze the relationship of its resonant frequency and the measured density, as shown in Figure 4. The curves were obtained from the results of coupling simulations and experiments when the microcantilever was vibrating in fluids with different densities under the first two resonant modes.

In Figure 4, the resonance frequency was varied linearly with the fluid density, but we know that the fluid density is indeed inversely proportional to the square of resonant frequency according to Equation (11). The Taylor expansion at *f*_fluid_ = *a* (*a* > 0) of Equation (11) can be written as:

(15) $$\rho_{f}=\frac{\rho_{f}{|}_{f_{\text{fluid}}=a}}{0!}+\frac{\rho_{f}^{\prime }{|}_{f_{\text{fluid}}=a}}{1!}(f-a)+\frac{\rho_{f}^{\prime \prime }{|}_{f_{\text{fluid}}=a}}{2!}{\left(f-a\right)}^{2}+...+\frac{\rho_{f}^{\left(n\right)}{|}_{f_{\text{fluid}}=a}}{n!}{\left(f-a\right)}^{n}+R_{n}(f)$$

where *R*_n_(*f*) is the high order infinitesimal of (*f − a*)^n^, and *R*_n_(*f*) approaching zero, thus Equation (15) can be simplified as follows:

(16) $$\rho_{f}=k_{1}a^{-2}+k_{2}+(-2k_{1}a^{-3})(f-a)+3k_{1}a^{-4}{\left(f-a\right)}^{2}+...$$

The resonant frequency of sensor in this paper are much larger than 4000 Hz, so the quadratic coefficient is very small due to the negative fourth power of *a*, and the coefficients of higher order term are all close to zero. Then, Equation (16) is changed as follows:

(17) $$\rho_{f}=-2k_{1}a^{-3}f+3k_{1}a^{-2}+k_{2}$$

Figure 4 showed that the slopes and linearities of the two kinds of results were fairly consistent. When the density of the fluid decreased, the resonant frequency increased with a linearity larger than 0.99. Also, the relative deviations of the resonant frequency in the simulation and experiment under the same vibration were smaller than 10%, which validated the correctness of the fluid-solid coupling simulation results. Therefore, the fluid-solid coupling simulation was very useful to guide the design of the microcantilever.

In addition, it’s shown that the slopes of the fitting straight lines under the second order mode were larger than those under the first order mode. This means the variation of resonant frequency under higher order mode was larger with the same fluid density fluctuation. Thus, the sensitivity of fluid density measurement was higher under the second order mode than that under the first order mode. The density sensitivity under the second order mode in this paper was about 2 times larger than that with the value of −2.6 Hz·(kg·m^−^^3^)^−^^1^ in [29].

## 3. Fabrication and Experiment

### 3.1. Microcantilever Design

**Figure 5:**
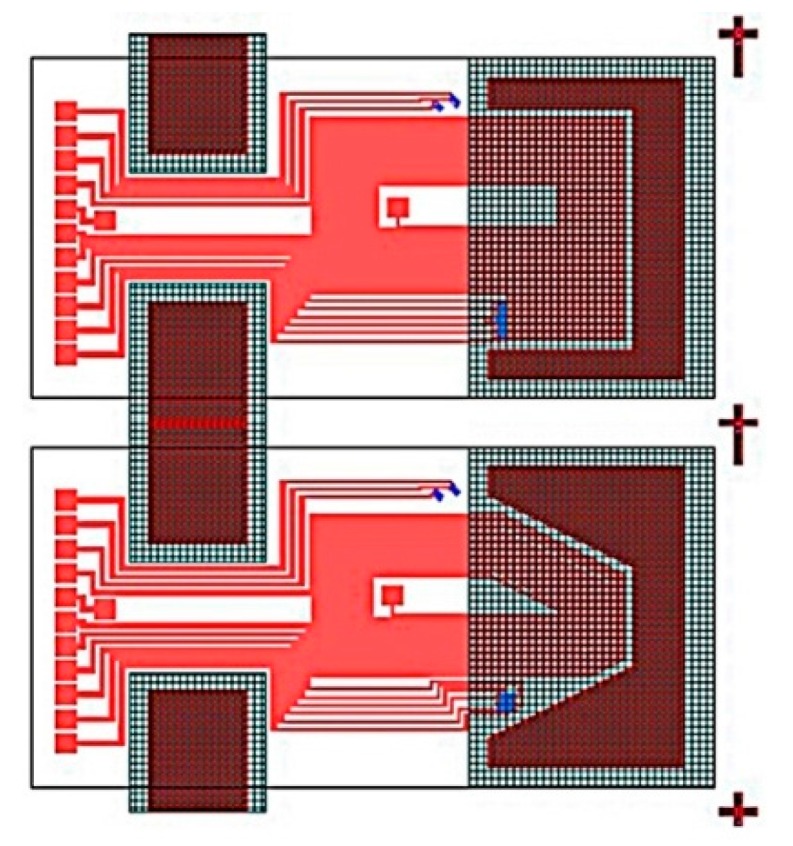
Two differentmicro-cantilever chips.

**Figure 6:**
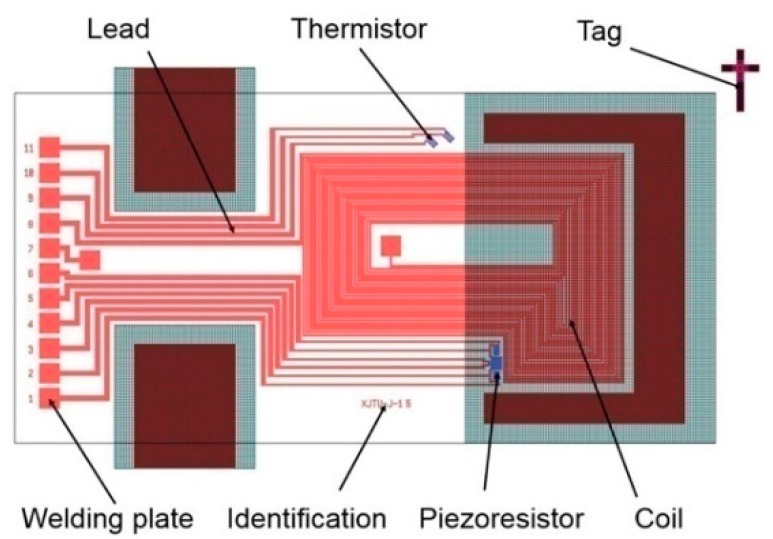
Layout of the rectangular microcantilever chip.

Two different micro-cantilever chips with rectangular and trapezoidal structures were designed according to the simulation results as shown in Figure 5 and Figure 6.

The sensing chip with the rectangular microcantilever was taken as an example to demonstrate the layout in Figure 6, where its dimensions were the same as in the simulation model. In the layout, there is a metal coil, four piezoresistors constituting the Wheatstone bridge, thermistor, identification tags, inner leads and welding pads. The sensing chip was fabricated using MEMS technology [30]. The microcantilever chip was excited to vibrate by the Lorentz force which was generated by the alternating current through the metal coil in the magnetic field. When the frequency of the alternating current was equal to the resonant frequency of the microcantilever chip, resonance would happen and the vibrating amplitude achieved its peak value. The amplitude data were obtained by the output of the Wheatstone bridge for the resistance values of piezoresistors were changed correspondingly. These data were fitted by the curves to calculate the resonant frequency and quality factor of the microcantilever chip. A thermistor was used to provide a reference temperature.

### 3.2. The Experimental System

**Figure 7:**
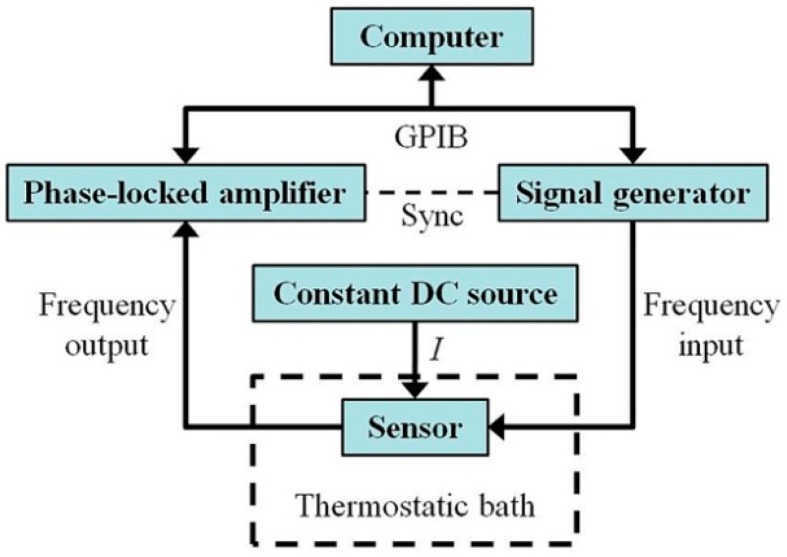
Block diagram of experimental system.

**Figure 8:**
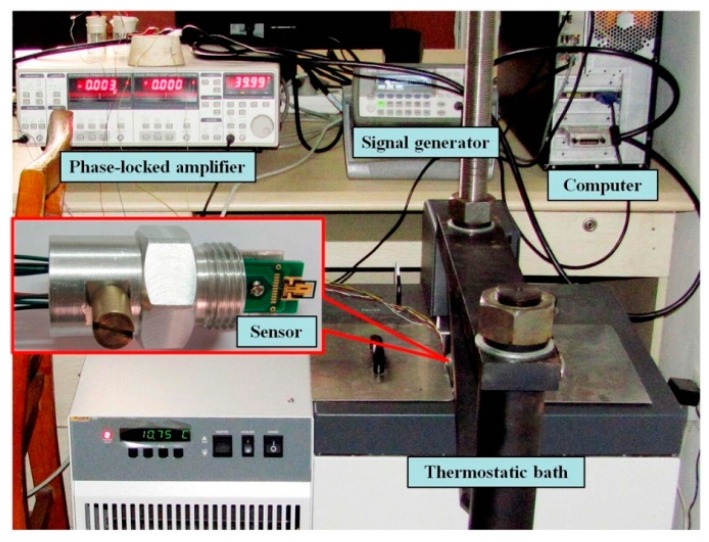
Experimental system.

The schematic diagram and object diagram of the experimental system are shown in Figure 7 and Figure 8. The resonant sensor was packaged with the developed sensing chip. The external magnetic field was provided by a samarium cobalt permanent magnet with a strength of 0.28T. The magnetic field direction was parallel to length direction of the microcantilever chip. A signal generator (33220 A Agilent, Santa Clara, CA, USA) provided a sinusoidal AC voltage with suitable amplitude and frequency to power the metal coil of the sensing chip. At the same time, it also provided a sync reference voltage signal to the phase-locked amplifier (SR830, Stanford, Sunnyvale, CA, USA). The first and second order modes were both realized by increasing the frequency of exciting signal (AC voltage through the coil) to be the first and second order resonant frequencies of the micro-cantilever. The Wheatstone bridge was powered by a constant current source with a value of 2 mA, and its output signal was detected by the Stanford SR830 to determine the vibration mode. The coupling of the AC voltage to the resistors of the Wheatstone bridge could be ignored because the resistors were located near the fixed end of the micro-cantilever and their displacements were very small. The signal generator and phase-locked amplifier were both controlled by a computer through the general purpose interface bus (GPIB) lines. The environment temperature was kept with different constant values by a thermostatic (7008, Fluke, Everett, WA, USA).

**Figure 9:**
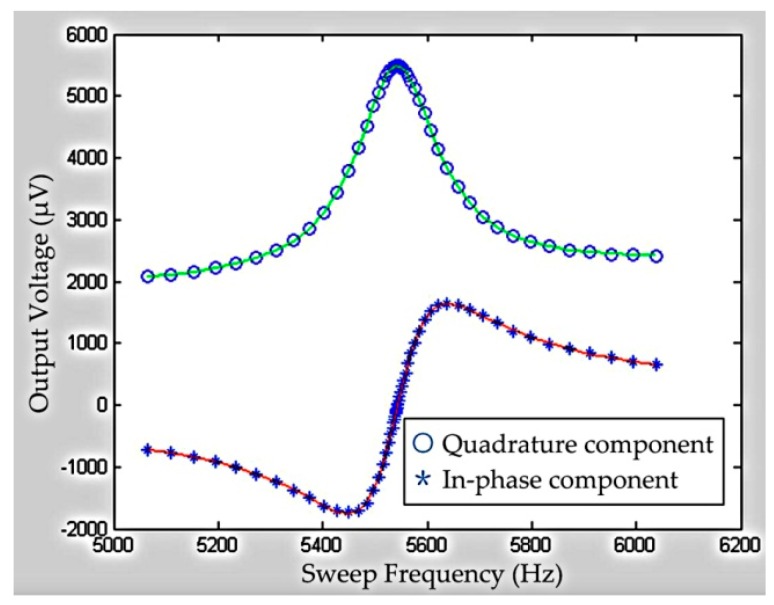
The resonant fitted curves.

The resonant frequency and half peak width should be obtained to calculate the fluid density and viscosity using Equations (11) and (14). The Stanford S830 detected the output signals of the Wheatstone bridge when the sinusoidal AC voltage excited the metal coil from low frequency to high frequency. The signal amplitude of the Wheatstone bridge could be intensified when the input frequency of AC voltage was close to the resonant frequency of the micro-cantilever chip in the fluid. The obtained data of the amplitude *versus* the sweeping frequency are shown in Figure 9. The data in blue * were the in-phase component of voltage amplitude, and the data in blue ○ were the quadrature component of voltage amplitude. All data were fitted by the MATLAB software to obtain the fitting equation. Then the resonant frequency, amplitude and half peak width of the resonant sensor could be calculated based on this equation.

## 4. Results and Discussion

In order to test the sensor’s performance, different fluids with various densities and viscosities should be measured by the proposed sensor. The measured fluids included *n*-pentane, *n*-hexane, *n*-heptane, isooctane, *n*-octane, 0.65 cs silicone oil, cyclohexane and methylbenzene. A specific fluids has different densities and viscosities under different temperatures. For the alkane fluids, their physical properties are the most stable from 20 °C to 25 °C. Their reference values of density, viscosity and sonic speed under standard atmospheric pressure and different temperature (293.15 K and 298.15 K) were calculated using the Reference Fluid Properties (REFPROP) software. In the field of thermophysical properties, the REFPROP software [31,32] is often used to provide the reference values of fluid density, viscosity, conductivity, and so on. Therefore, we used the reference values from the software to estimate the accuracy of each measurement.

**Figure 10:**
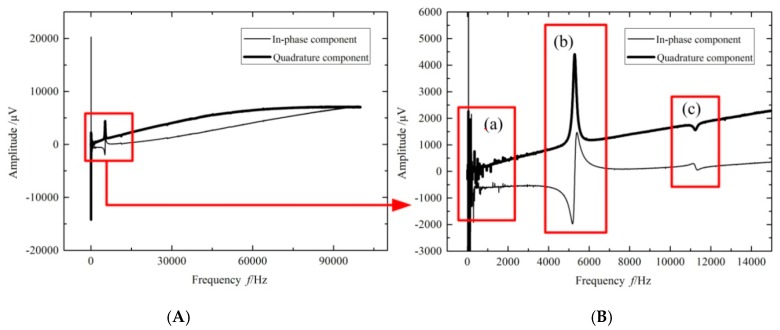
Sweep frequency results of rectangular micro-cantilever in 0.65 cs silicone oil. (**A**) The curve of sweep frequency; (**B**) The detail of red box in picture (**A**): The red box (a) is the noise below 2 kHz; (b) is the first order resonance (fFlexure); (c) is the second order resonance (tTorsion).

For the rectangular microcantilever chip, a frequency swept curve was obtained in 0.65 cs silicone oil under different resonant modes, as shown in Figure 10. There were many irregular peaks in the curve below 2 kHz as shown in Figure 10Ba because the microcantilever chip was easily disturbed by external vibration and noise. 

It was easy to make sure that this curve had two resonant peaks in the frequency range from 1 to 100 kHz. In theory, another resonant peaks should also appear in the frequency range from 1 to 100 kHz, but the strains of the piezoresistors in the microcantilever chip were so small under higher order resonant modes, so the Wheatstone bridge’s outputs were also small and difficult to detect. The vibration modes could be estimated by the coupled fluid-solid simulation results. For example, Figure 10Bb,c show the resonant curves under first order flexural vibration and first order torsional vibration, respectively. Therefore, the fluid viscosity and density could be measured under the first two order modes, and the experimental results were discussed as follows: the measurement data of the rectangular microcantilever chip under flexural resonant mode and torsional resonant mode are shown in Table 1 and Table 2, respectively. The values of *Q* were calculated by *Q* = *f*_fluid_/2*g*_fluid_ using the MATLAB fitting method. The reference values of *ρ*_ref_ and *η*_ref_ for the fluid density and viscosity were obtained from the REFPROP software.

**Figure 11:**
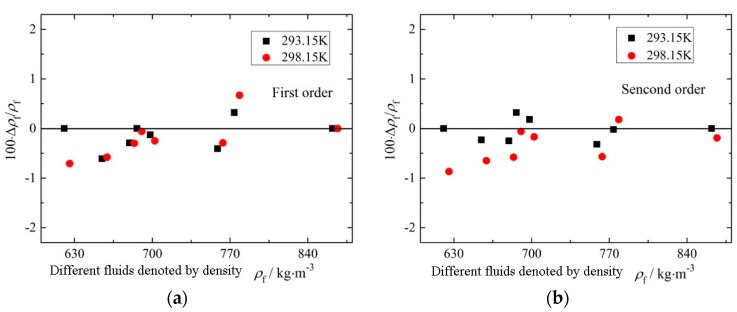
Relative errors of density under different order modes. (**a**) Under first order mode (flexure); (**b**) under second order mode (torsion).

**Figure 12:**
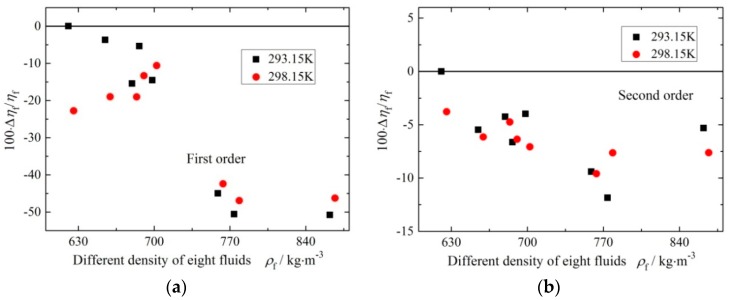
Relative errors of viscosity under different order modes. (**a**) Under first order mode (flexure); (**b**) under second order mode (torsion).

Here only experimental results and data acquisition method of the rectangular microcantilever chip were discussed. The deviations between the experimental values and reference values of the measured densities and viscosities under different resonant modes are shown in Figure 11 and Figure 12. In addition, the measurement results of the trapezoidal micro-cantilever chip were obtained similarly, and for brevity are not repeated in this paper.

**Figure 13:**
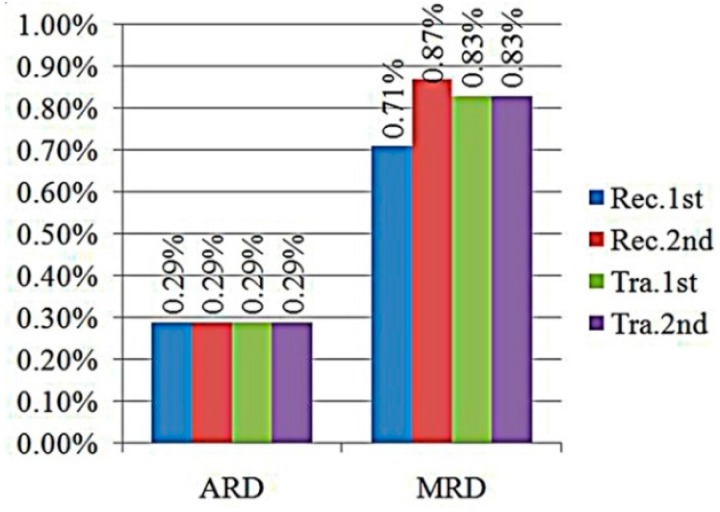
Deviations of density values.

**Figure 14:**
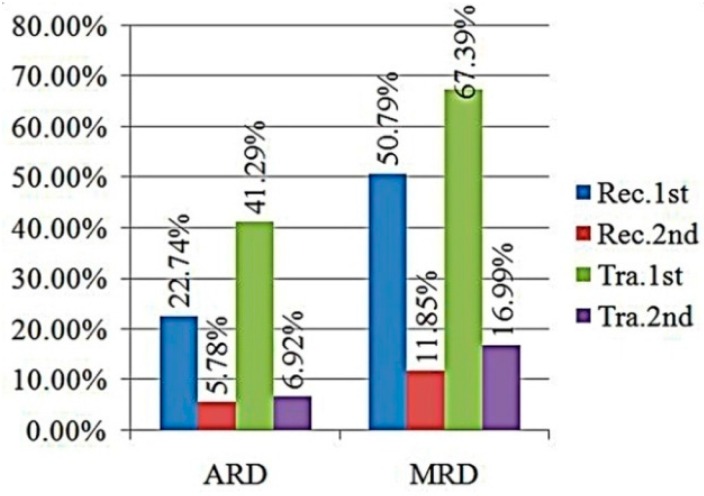
Deviations of viscosity values.

In order to describe and evaluate the measurement results of fluid density and viscosity, the average relative deviation (ARD) and maximum relative deviation (MRD) were introduced to analyze the measurement accuracy. The ARD is the average relative errors of all corresponding measurement results of the eight fluids at two temperature points. The MRD is the maximum relative deviation of all corresponding measurement results of eight fluids as well. Figure 13 and Figure 14 showed measurement accuracies of density and viscosity in ARD and MRD under different resonant modes.

As shown in Figure 11 and Figure 13, the fluid density range was from 620.83 kg/m^3^ to 866.87 kg/m^3^. In Figure 13, all ARDs in the density were approximate and all less than 0.3%, even under different resonant modes. In addition, the MRDs in the density were all less than 1% and also did not changed too much under different resonant modes. Therefore, the chip shape and the resonant modes had only slight influences on the fluid density measurement accuracy.

In Figure 12 and Figure 14, the fluid viscosity range was from 217.9 μPa·s to 961.8 μPa·s. The ARDs in the viscosity between experimental values and reference values were 22.74% and 41.29% under first order resonant mode for the rectangular and trapezoidal micro-cantilevers, respectively. However, the ARDs in the viscosity between experiment values and reference values under the second order resonant mode were 5.78% and 6.92% for them, respectively. The ARDs of the fluid viscosity measurement were larger under the flexural mode because the quality factors of the microcantilever chip were fairly small under the first resonant mode. The ARDs of viscosity measurement under the torsional mode were about 10% and could be accepted in comparison with other viscosity measuring methods [14,15,33,34]. It meant that the accuracy of fluid viscosity could be enhanced using the torsional resonant mode due to the higher quality factors as shown in Table 1 and Table 2.

## 5. Conclusions

In fluid parameter measurement, the main factor affecting the resonance frequency of a resonant sensor was the fluid density, and the main factor affecting the resonance amplitude was the fluid viscosity. In this paper, a fluid density measuring equation had been deduced from the resonant frequency of a microcantilever, and the fluid viscosity measuring equation had been deduced from the quality factor. In this research a simulation method was built to guide the design of a microcantilever resonant chip. An experimental system had been set up for the fabricated MEMS resonant sensor to measure the densities and viscosities of different fluids. The experimental results showed that the fluid density measurement accuracy could not be improved by using microcantilever chips of different shapes or high order resonant mode, but the fluid viscosity measurement accuracy under the torsional vibration mode was obviously better than that under the flexural vibration mode due to the higher quality factors under the former mode. Therefore, it’s necessary to investigate methods to improve the quality factor such as optimizing coil pattern and piezoresistors location or selecting a high order resonant mode.

## Figures and Tables

**Table 1:** Experimental results of rectangular micro-cantilever chip under flexural resonant mode.

Fluid	*T* (K)	*f*_fluid_ (Hz)	*Q*	*ρ*_ref_ (kg·m^−3^)	*ρ*_f_ (kg·m^−3^)	*η*_ref_ (μPa·s)	*η*_f_ (μPa·s)
*n*-pentane	293.15	5737.16	38.18	625.75	621.32	227.5	175.7
	298.15 ^a^	5738.98	34.75	620.83	620.83	217.9	217.9
*n*-hexane	293.15	5615.55	32.68	659.36	655.55	312.3	253
	298.15	5632.09	30.95	654.78	650.76	296.3	285.4
*n*-heptane	293.15	5527.5	29.01	683.82	681.75	411.4	333.1
	298.15	5541.15	29.17	679.6	677.6	388.5	328.5
isooctane	293.15	5500.02	25.63	690.6	690.18	506.1	438.6
	298.15	5512.56	25.25	686.3	686.32	478.6	452.8
*n*-octane	293.15	5466.83	24.53	702.29	700.54	542	484.6
	298.15	5476.98	25.74	698.27	697.35	509.7	435.7
silicone oil (0.65 cs)	293.15	5283.39	27.41	763.61	761.36	672.3	387.1
	298.15	5299.43	28.39	758.87	755.79	650	357.5
cyclohexane	293.15	5220.12	24.24	778.63	783.84	961.8	510.5
	298.15	5240.91	25.99	773.89	776.36	884.7	437.3
methylbenzene	293.15	5004.75	30.48	866.87	866.85	588	316
	298.15 ^b^	5016.13	32.48	862.2	862.2	556	273.6

Note: The groups of data which have superscripts of a and b are used to calibrate the constants in the Equations (11) and (14). *k*_1_, *k*_2_, *k*_3_ and *k*_4_ were 2.5729154926 × 10^10^, −160.36, 3.9768556044 × 10^13^ and 3.777 × 10^−3^, respectively.

**Table 2:** Experimental results of rectangular micro-cantilever chip under torsional resonant mode.

Fluid	*T* (K)	*f*_fluid_ (Hz)	*Q*	*ρ*_ref_ (kg·m^−3^)	*ρ*_f_ (kg·m^−3^)	*η*_ref_ (μPa·s)	*η*_f_ (μPa·s)
*n*-pentane	293.15	12,210.45	75.63	625.75	620.32	227.5	218.9
	298.15 ^a^	12,206.51	75.79	620.83	620.83	217.9	217.9
*n*-hexane	293.15	11,951.80	66.11	659.36	655.08	312.3	293.1
	298.15	11,964.81	67.54	654.78	653.28	296.3	280.1
*n*-heptane	293.15	11,777.05	57.71	683.82	679.87	411.4	391.9
	298.15	11,790.67	59.15	679.6	677.89	388.5	372.0
isooctane	293.15	11,706.48	52.73	690.6	690.19	506.1	473.9
	298.15	11,717.74	54.24	686.3	688.53	478.6	446.8
*n*-octane	293.15	11,633.45	51.28	702.29	701.08	542	503.7
	298.15	11,643.80	51.99	698.27	699.52	509.7	489.4
silicone oil (0.65 cs)	293.15	11,264.99	54.17	763.61	759.26	672.3	609.2
	298.15	11,282.02	54.63	758.87	756.45	650	587.6
cyclohexane	293.15	11,141.85	39.38	778.63	780.01	961.8	888.3
	298.15	11,178.47	41.91	773.89	773.77	884.7	779.9
methylbenzene	293.15	10,675.39	50.59	866.87	865.21	588	543.2
	298.15 ^b^	10,690.88	51.33	862.2	862.20	556	526.5

Note: The groups of data which have superscripts of a and b are used to calibrate the constants in the Equations (11) and (14). *k*_1_, *k*_2_, *k*_3_ and *k*_4_ were 1.1844404355 × 10^11^, −174.10, 1.567181537564 × 10^15^ and 6.649 × 10^−4^, respectively.
